# Supplementary material for: Identification of photoperiod-induced specific miRNAs in the adrenal glands of Sunite sheep (Ovis aries)
Source: Front Vet Sci. 2022 Jul 22;9:888207. doi: 10.3389/fvets.2022.888207 (PMC9354845; doi:10.3389/fvets.2022.888207)
Supplement: Supplementary file 1 [file Data_Sheet_1.ZIP › Supplementary file/miRDeep2 file.zip!/miRDeep2 file/Novel_Structure/SP42A3/Novel_176.pdf]

| | |
|---|---|
| Provisional ID | : NC_019484.2_32673 |
| Score total | : 26.4 |
| Score for star read(s) | : 3.9 |
| Score for read counts | : 18.5 |
| Score for mfe | : 2.4 |
| Score for randfold | : 1.6 |
| Score for cons. seed | : |
| Total read count | : 48 |
| Mature read count | : 47 |
| Loop read count | : 0 |
| Star read count | : 1 |

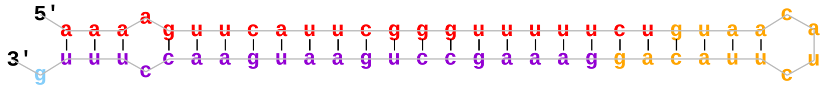


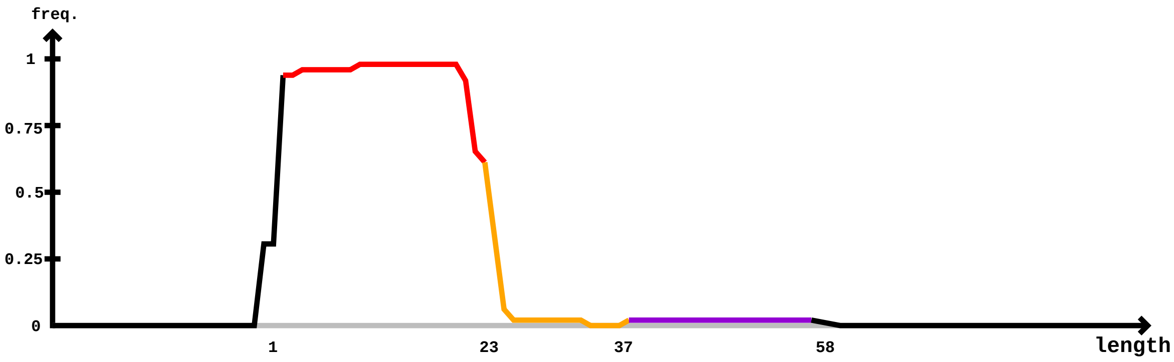


Mature Star

```
5'- uccccacauauuggguuggcugaaaaguucauucggguuuuucuguaacaucuuacaggaaagccugaaugaaccuuuggccaacccaauauuuccgaccaguugcaagggc  -3'   obs
    uccccacauauuggguuggcugaaaaguucauucggguuuuucuguaacaucuuacaggaaagccugaaugaaccuuuggccaacccaauauuuccgaccaguugcaagggc         exp
    .......((((((((((((((((..(((((((((((((((((((((((....))))))))))))))))))))))).))))))))))))))))......((........))..   reads   mm   sample
    ....................ugaaaaguucauucggguuuu.......................................................................   3       0    seq
    ....................ugaaaaguucauucggguuuuu......................................................................   12      0    seq
    ......................aaaaguucauucggguuuuu......................................................................   1       0    seq
    ......................aaaaguucauuAggguuuuuc.....................................................................   2       1    seq
    ......................aaaaguucauuAggguuuuucu....................................................................   27      1    seq
    ......................aaaaguucauuAggguuuuucugu..................................................................   1       1    seq
    ........................aaguucauuUggguuuuucugu..................................................................   1       1    seq
    ..............................auuAggguuuuucuguaacaucuu..........................................................   1       1    seq
    ..........................................................gaaaAccugaaugaaccuuu..................................   1       1    seq
```
